# Serum Metabolomic Signatures in Nonhuman Primates Treated with a Countermeasure and Exposed to Partial- or Total-Body Radiation

**DOI:** 10.3390/metabo15080546

**Published:** 2025-08-12

**Authors:** Alana D. Carpenter, Yaoxiang Li, Benjamin E. Packer, Oluseyi O. Fatanmi, Stephen Y. Wise, Sarah A. Petrus, Martin Hauer-Jensen, Amrita K. Cheema, Vijay K. Singh

**Affiliations:** 1 Division of Radioprotectants, Department of Pharmacology and Molecular Therapeutics, F. Edward Hébert School of Medicine, Uniformed Services University of the Health Sciences, Bethesda, MD 20814, USA; alana.carpenter.ctr@usuhs.edu (A.D.C.); benjamin.packer.ctr@usuhs.edu (B.E.P.); oluseyi.fatanmi@usuhs.edu (O.O.F.); stephen.wise.ctr@usuhs.edu (S.Y.W.); sarah.petrus.ctr@usuhs.edu (S.A.P.); 2 Armed Forces Radiobiology Research Institute, Uniformed Services University of the Health Sciences, Bethesda, MD 20814, USA; 3 Department of Oncology, Lombardi Comprehensive Cancer Center, Georgetown University Medical Center, Washington, DC 20057, USAamrita.cheema@georgetown.edu (A.K.C.); 4 Division of Radiation Health, Department of Pharmaceutical Sciences, University of Arkansas for Medical Sciences, Little Rock, AR 72205, USA; mhjensen@uams.edu; 5 Department of Biochemistry, Molecular and Cellular Biology, Georgetown University Medical Center, Washington, DC 20057, USA

**Keywords:** GT3, biomarkers, metabolomics, nonhuman primates, radioprotection, partial-body irradiation, total-body irradiation

## Abstract

**Background**: Irradiation-induced injury is a common fallout of radiological/nuclear accidents or therapeutic exposures to high doses of radiation at high dose rates. Currently, there are no prophylactic drugs available to mitigate radiation injury as a result of exposure to lethal doses of ionizing radiation. Gamma-tocotrienol (GT3) of vitamin E is a promising radioprotector under advanced development which has been tested for efficacy in both murine and nonhuman primate (NHP) models. Previously, we have demonstrated that GT3 has radioprotective efficacy in intestinal epithelial and crypt cells, and restores transcriptomic changes in NHPs with a supralethal dose of 12 Gy total-body irradiation (TBI). **Methods**: In this study, we evaluated the effect of 12 Gy partial-body irradiation (PBI) or TBI on metabolomic changes in serum samples and the extent to which GT3 was able to modulate these irradiation-induced changes. A total of 32 nonhuman primates were used for this study, and blood sample were collected 3 days (d) prior to irradiation, and 4 h, 8 h, 12 h, 1 d, 2 d, and 6 d post-irradiation. **Results**: Our results demonstrate that exposure to a supralethal dose of radiation induces a complex range of metabolomic shifts with similar degrees of dysregulation in both partial- and total-body irradiated animals. The C21-steroid hormone biosynthesis and metabolism pathway was significantly dysregulated in both PBI and TBI groups, with minimal protection afforded by GT3 administration. **Conclusions**: GT3 offered a differential response in terms of protected metabolites and pathways in either group that was most effective at the early post-irradiation time points.

## 1. Introduction

Individuals exposed to radiation are at risk of developing life-threatening complications. Acute exposure to radiation can lead to radiation sickness, also known as acute radiation syndrome (ARS), which consists of a constellation of health effects that can impact multiple tissues and organs. The lymphatic (specifically bone marrow), gastrointestinal, pulmonary, central nervous, and cutaneous systems are very sensitive to irradiation [1,2,3]. Clinical indications of ARS manifest as the hematopoietic (H-ARS; 2–6 Gy), gastrointestinal (GI-ARS; 6–8 Gy), and neurovascular (NV-ARS; >8 Gy) subsyndromes [4,5]. Individuals exposed to radiation doses resulting in H-ARS or GI-ARS are expected to benefit from treatment with radiation medical countermeasures (MCMs). There are nine United States Food and Drug Administration (US FDA)-approved MCMs for the mitigation of ARS: Neupogen, Neulasta, Leukine, Nplate, and five biosimilars of Neupogen and Neulasta (Nypozi, Zarxio, Udenyca, Stimufend, and Ziextenzo) [6,7,8,9,10,11,12,13,14,15]. All nine agents are only indicated for H-ARS and are only effective when administered soon after radiation exposure, stored in a cold chain, and generally administered in a hospital setting. Given the events unfolding in the current conflicts in Eastern Europe and the Middle East, the development of an effective prophylactic radiation countermeasure would be an invaluable asset for military and emergency preparedness, significantly enhancing the ability to protect responding personnel from the harmful effects of radiation exposure in the event of nuclear accidents, radiological emergencies, or acts of warfare.

During radiological emergencies, it is crucial to determine the extent of radiation exposure to provide timely and appropriate medical intervention [16,17,18]. Physical and biological dosimetry can be used in combination to determine the radiation exposure dose, which accelerates clinical evaluation and response [19,20]. The gold standard for biological dosimetry includes cytogenic analysis of peripheral blood lymphocytes, dicentric chromosome assay, and other chromosomal aberration assays. However, these methods are labor-intensive and difficult for population-based screening during a mass casualty scenario [21]. Hence, it is imperative to develop accurate and dependable methods suitable for mass screening to triage radiation-exposed individuals for medical care. Assays involving genomic, proteomic, and metabolomic markers have the potential for rapid high-throughput screening of masses and can reliably account for population variability, but of these methods the most promising approach for assessing these measures are metabolomic and lipidomic profiling [22,23,24,25]. Metabolomics is an emerging field that allows researchers to investigate downstream changes in gene expression or alterations by qualitatively and quantitatively analyzing metabolites present in biological samples by mass spectrometry (MS), often coupled with liquid or gas chromatography. These identified metabolites may serve as biomarkers that are important for MCM development, particularly for dose conversions from animal models to humans, a critical step for MCM development as specified by the US Food and Drug Administration (FDA) Animal Rule [26,27,28].

One of these radiation MCMs under development is gamma-tocotrienol (γ-tocotrienol; GT3), a potent antioxidant and isomer of vitamin E that is currently under advanced development as a radioprotector for pre-exposure prophylaxis [29,30,31]. In addition to its strong antioxidant activity, GT3 exhibits significant anti-inflammatory effects and neuroprotective properties, making it a promising potential radioprotectant. It has been studied in rodents and NHPs and has demonstrated radioprotective efficacy when administered 24 h before TBI. GT3 has also been shown to induce high levels of granulocyte colony-stimulating factor (G-CSF), mobilize progenitors, and enhance the radioprotective efficacy of low doses of amifostine [32]. These initial findings prompted us to initiate advanced, large animal studies using NHPs [31]. We have also conducted studies using multiomic platforms to identify biomarkers for radiation injury which will be helpful to obtain regulatory approval of MCMs following the US FDA Animal Rule [28,33,34]. It is important to note that a single injection of GT3 24 h prior to radiation exposure without any supportive care (blood products) was equivalent, in terms of improving hematopoietic recovery, to multiple doses of Neupogen and two doses of Neulasta with full supportive care (including blood transfusions) in the NHP model [7,13,31]. Additionally, GT3 is a radioprotector which can be administered prior to exposure as a prophylaxis, can be stored at ambient temperature, and will not need to be administered in a clinical setting. Recently, we investigated the radioprotective efficacy, transcriptomic changes, and histopathological recovery of GT3 using supralethal doses of 12 Gy PBI and TBI in an NHP model [35,36].

In this study, we profiled metabolomic changes in the serum samples of rhesus macaques exposed to a supralethal dose of 12 Gy partial- or total-body radiation capable of inducing severe ARS. Furthermore, we investigated the restorative changes to metabolomic profiles induced by the promising radiation countermeasure, GT3. We posited that metabolic alterations caused by partial- or total-body exposure will likely be reversed, at least in part, upon GT3 treatment. To assess the radioprotective effects of GT3, therefore, metabolite significance in both the radiation and GT3 effects comparisons was analyzed, in addition to the change in directionality due to GT3 treatment. GT3 restored irradiation-induced changes to metabolomic profiles to a limited extent. This may be due to the limitation of GT3’s efficacy at high radiation doses inducing GI-ARS. Ultimately, the targeted dose of 12 Gy may be somewhat more lethal than our expectation.

## 2. Materials and Methods

### 2.1. Experimental Design

**Figure 1:**
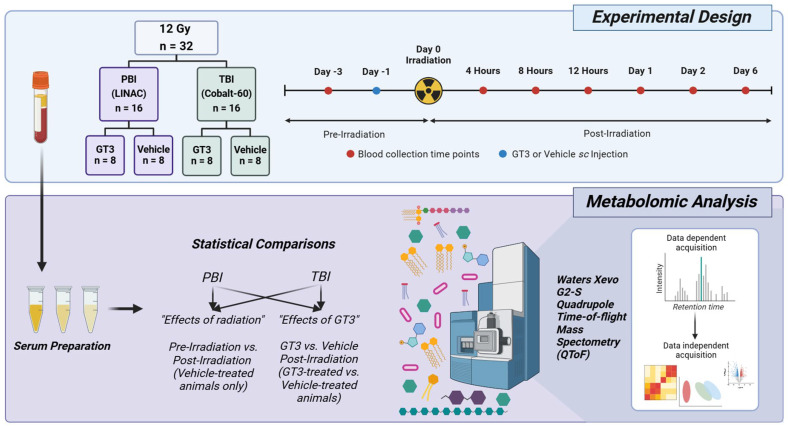
Overall experimental design and metabolomics workflow for this study to assess the effect of 12 Gy partial- or total-body irradiation and GT3 administration on serum metabolomic profiles. Figure was created by the authors with BioRender.com.

The primary scope of this study was to assess gastrointestinal (GI) injury in animals exposed to 12 Gy partial- or total-body radiation. Power analysis was performed to determine the appropriate sample size needed to detect changes with a statistical power of greater than 80% and a significance level of below 0.05, ensuring the study was both scientifically valid and ethically justified. Based on a two-sided two-sample *t*-test (α = 0.05), achieving 80% power with eight animals per group requires a standardized effect size (Cohen’s d) of approximately 1.51; under this assumption, a sample size of eight NHPs per arm provides the intended 80% statistical power. A total of 32 NHPs (rhesus macaques) were used for this study; 16 animals were exposed to partial-body radiation (LINAC X-ray radiation, 1.3 Gy/min) and the remaining 16 animals were exposed to total-body radiation (cobalt-60 γ-radiation, 0.6 Gy/min). In each group, eight NHPs were administered a 37.5 mg/kg dose of GT3 via subcutaneous (*sc*) injection, and eight were administered the corresponding vehicle via the same route 24 h prior to radiation exposure. Euthanasia was scheduled for animals on day (d) 4 and 7 post-irradiation, at which time specialized tissue collection was performed to assess GI injury [35,36]. Blood samples were also collected throughout the course of the study, and various omics studies were performed to assess radiation-induced changes and any protective effects offered by GT3. In detail, eight samples were collected from vehicle or GT3-treated animals on d −3, 4 h, 8 h, 12 h, d 1, and d 2. Due to the scheduled euthanasia of animals on d 4, only five samples were collected from each group on d 6. In total, 104 samples were analyzed for the PBI group, while 106 samples were analyzed for the TBI group. Insufficient serum volume prevented two samples from being analyzed in the PBI group. In an effort to refer to each comparison succinctly, the comparison between pre-irradiation and the vehicle/irradiated group is referred to as the “radiation effects” comparison, while the comparison between the GT3-treated and vehicle/irradiated group is referred to as the “GT3 effects” or “drug effects” comparison. Overall experimental design and metabolomics workflow can be viewed in Figure 1.

### 2.2. Animals

A total of 32 rhesus macaques (*Macaca mulatta*, Chinese sub-strain, 14 males and 18 females, 3.5–5.5 years of age, weighing 3.84–5.6 kg) were used in this study. The 32 animals were obtained from the National Institutes of Health Animal Center (NIHAC, Poolesville, MD, USA) or from an approved NHP vendor (PrimeGen, Hines, IL, USA). All animals were maintained at Bioqual, Inc. (Rockville, MD, USA), a facility accredited by the Association for Assessment and Accreditation of Laboratory Animal Care (AAALAC)-International. Prior to the experiment, all NHPs underwent a quarantine period of six weeks. During the quarantine period, animals were assigned to GT3 and vehicle-treated groups after being divided by sex and body-weight. Due to study-specific reasons, paired housing was not possible; housing the animals individually ensured the prevention of conflict injuries, and as irradiated animals are more prone to infection due to immune system suppression, this was a vital course of action in order to obtain unobstructed data. Other details about animal feed and upkeep are described earlier [31]. All animal procedures were approved (Protocol # 18-060 approved on 27 June 2018) by the Institutional Animal Care and Use Committee (IACUC, Bioqual, Inc.) and the Department of Defense Animal Care and Use Review Office. The Guide for the Care and Use of Laboratory Animals was strictly adhered to throughout this study [37]. This study is reported in accordance with ARRIVE guidelines.

### 2.3. Drug Preparation and Administration

GT3 (50 mg/mL) and the olive oil-based vehicle formulation were acquired from Callion Pharma, LLC (Jonesborough, TN, USA). At least 48 h prior to drug injections, the area between the shoulder blades (dorsal scapular area) was shaved so that the skin could be easily observed for any adverse reactions such as rash/eruption, inflammation, irritation, and abscess formation following GT3 or vehicle administration [36]. To prepare for drug administration, the injection site was wiped with 70% isopropyl rubbing alcohol and allowed to air dry. Once dried, the drug or vehicle was administered *sc* using a 3 mL disposable luer-lock syringe with a 25-gauge 5/8-inch needle. A 37.5 mg/kg dose was administered to each animal based on individual body weights.

### 2.4. Radiation Exposure

The initial objective of this study was to investigate the effect of GT3 in NHPs exposed to a radiation dose inducing GI-ARS, and hence 12 Gy partial- and total-body radiation exposures were used. We have reported results with GI tissues and transcriptomics earlier [35,36,38,39]. From the same animal samples, this study was conducted to investigate the metabolomic changes. It is important to note that the dose rates for PBI (1.3 Gy/min) and TBI (0.6 Gy/min) differed due to the specific experimental conditions required for NHP irradiations. For PBI specifically, the field size to fit the head-to-knee length of each animal in a uniform radiation field and the resulting distance from the source yielded a dose rate of 1.3 Gy/min. In contrast, the dose rate of 0.6 Gy/min is the standard dose rate for TBI conducted by our group and was used in this study to maintain consistency and reliability of inter-experiment comparisons.

#### 2.4.1. Partial-Body Radiation Exposure

NHPs were exposed to a 12 Gy partial-body radiation dose using a 4 MV photon beam from an Elekta Infinity clinical linear accelerator (LINAC) with a dose rate of approximately 1.3 Gy/min. As radiation exposure can often result in nausea and vomiting, all animals were fasted for approximately 12–18 h prior to their scheduled exposures. Digital calipers were used to measure the location of the absorbed dose target (the abdominal anterior/posterior lengths) for each individual NHP. Additional measurements were also performed, including distances from the crown of the head to the hip, knee, and foot to aid in the accurate measurement for bone marrow sparing. After the animals were brought to the Armed Forces Radiobiology Research Institute (AFRRI) for irradiation, they were transferred from their transport cages into temporary housing cages. In preparation for irradiation, the identity of each animal was confirmed using their unique tattooed ID numbers. Using the cage’s squeeze-back mechanism, the NHPs were administered 10–15 mg/kg of ketamine hydrochloride (Zoetis, Inc., Kalamazoo, MI, USA; 100 mg/mL) through an intramuscular (*im*) injection for sedation. After confirming that the animal was completely sedated, NHPs were individually positioned on a custom restraint device mounted to the LINAC couch. After placing the animal in a supine position, restraints were used to fix the NHP’s limbs to the platform to limit possible movement in the event that the sedation began to wean. In order to spare 5% of the bone marrow, the field positioning was adjusted to extend from the top of the skull to the knee, excluding the tibia, ankles, and feet of the animals from radiation exposure, having a diagonal length of approximately 80 cm with a collimator angle of 45 degrees. Two gantry angles, 0° and 180°, were used to deliver half of the total radiation dose each; delivering the dose along both the anterior–posterior and posterior–anterior directions ensured that the absorbed radiation dose is homogeneous. An Advisor vital signs monitor (Smiths Medical, Dublin, OH, USA) was used to continuously monitor heart rate and temperature throughout the duration of the procedure. Additional details describing the PBI procedure, including LINAC calibration and dosimetry, can be found in an earlier publication [35,36].

#### 2.4.2. Total-Body Radiation Exposure

The high level ^60^Co gamma irradiator was used for TBI. To minimize the occurrence of irradiation-induced vomiting, all animals fasted for approximately 12–18 h prior to their scheduled exposures. Upon arrival to AFRRI, the animals were transferred into temporary housing cages. Approximately 15 min prior to irradiation, the unique tattoo identification numbers on the animals were confirmed, and the NHPs were sedated with an *im* injection of 10–15 mg/kg of ketamine hydrochloride (Zoetis, Inc., Kalamazoo, MI, USA; 100 mg/mL) using the cage’s squeeze-back mechanism. To deliver the exact radiation dose to each animal, the abdominal lateral separations of the animals at the height of the dose prescription point (core of the abdomen) were measured with a digital caliper approximately one week prior to irradiation. Animals were paired for irradiation based on the similarity of their lateral separations (+/−1 cm). If animals were not within 1 cm of another’s measurements, they were irradiated separately. These measurements were also used to ensure an accurate field of exposure by the facility dosimetrist [35]. Once anesthetized, the animals were secured in custom-made Plexiglas restraint boxes to maintain the proper positioning throughout the irradiation procedure and to limit the possibility of significant movement. Rope restraints were tethered from the NHP’s limbs to a cleat to secure them to the box [36]. As higher doses of radiation require longer exposure times, a 0.1–0.3 mL *im* booster of ketamine hydrochloride (100 mg/mL) was administered to the NHPs prior to the procedure (if deemed necessary) to limit movement while being irradiated. After being positioned facing away from each other on the irradiation platform, the two NHPs were exposed to a bilateral midline dose of 12 Gy at a dose rate of 0.6 Gy/min. Once irradiation was complete, animals were returned to the transport cart and to their cages in the holding area. They were then evaluated for complete recovery from the sedatives and irradiation procedure. Additional details of TBI and dosimetry are described in previous publications [35,36].

### 2.5. Blood Sample Collection

Blood was collected from the saphenous vein in serum-separating tubes, allowed to clot for at least 30 min, and centrifuged for 10 min at 400× *g*. Serum was isolated and stored at −70 °C until analysis [31].

### 2.6. Sample Preparation and Metabolomic Analyses

Serum was separated from whole blood using serum-separator tubes. After allowing blood to clot for 30 min, samples were centrifuged to isolate the serum, which was then placed into a new tube and stored at −80 °C until analysis. Metabolomic profiling was performed using UPLC-QTOF-MS. Metabolites were extracted from serum using a solvent mixture composed of methanol, isopropanol, water, and internal standards, followed by protein precipitation with acetonitrile. Centrifugation was performed to collect supernatants for analysis. Samples were then run on a UPLC system coupled to a Xevo G2 QTOF mass spectrometer using either a BEH or CSH C18 column for metabolomics or lipidomics, respectively. Mass spectrometry was performed in both positive and negative ionization modes, with regular injections of pooled quality control samples to monitor data consistency and instrument performance. Additional details for metabolomics analysis can be viewed in previous publications [40].

### 2.7. Statistical Analyses

Data acquired from the UPLC-TOFMS was processed with the XCMS R package (version 4.4.1) (Scripps Research Institute, La Jolla, CA, USA) to produce a comprehensive metabolic matrix containing retention time, mass to charge (*m*/*z*) values, and ion intensities. The data were normalized using internal standard normalization and QC-RLSC normalization, followed by log transformation and Pareto scaling prior to multivariate statistical analysis, which was conducted and visualized with custom R scripts [41]. Significantly dysregulated metabolites were identified by comparing normalized intensity values (*m*/*z* features) between GT3-treated and vehicle-treated NHPs at each of the seven post-irradiation time points using two-sided Mann–Whitney U tests. Because each animal contributed one sample per time point, we performed a separate nonparametric comparison at each time point rather than assuming a specific distribution across repeated measures. To control for multiple hypothesis testing across all *m*/*z* features and time points, *p*-values were adjusted using the Benjamini–Hochberg false discovery rate (FDR) procedure, with FDR < 0.05 considered significant. All statistically significant *m*/*z* values were then putatively annotated via METLIN [42], CEU Mass Mediator [43], and HMDB databases [44] for downstream pathway analysis. An all-inclusive list of metabolites assessed is provided in Supplementary Tables S1 and S2. Summaries of the statistical analyses performed for PBI and TBI are shown in Supplementary Tables S3 and S4. Fold changes (FC) are also included, which assess metabolite expression between the various treatment groups. Log2(FC) calculations were also performed, which enhance interpretability of these values and allow for easy integration into various data visualization formats. Additionally, the Python package Mummichog (version 2.0.6) was employed to identify disrupted metabolic pathways based on alterations in metabolite concentrations. These findings, including *p*-values and false discovery rates (FDR), are presented in Supplementary Tables S5 and S6. 

## 3. Results

**Figure 2:**
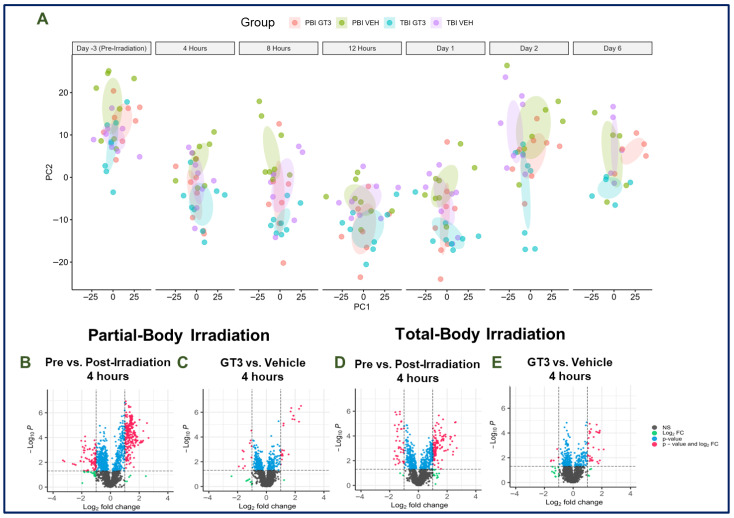
(**A**). Two-dimensional (2D) principal component analyses (PCA) plots showing overall group separation at each time point. Data was obtained in the electrospray negative ionization mode. Volcano plots comparing NHP serum profiles 4 h post partial-body irradiation (**B**,**C**) or post total-body irradiation (**D**,**E**).

Both PBI and TBI induced significant metabolomic perturbations in the serum samples collected from the irradiated animals (Figure 1). Interestingly, the number of significantly dysregulated pathways and metabolites was greater in PBI animals compared to TBI animals. Principal component analysis (PCA) plots were constructed to visualize overall group separation between radiation exposure types (PBI or TBI) and treatments (GT3 or vehicle) (Figure 2A). Overall, radiation response was similar between radiation exposure types and treatment groups, as suggested by the clustering in the PCA plots. Both PBI and TBI groups experienced a majority upregulation in response to irradiation, while a majority downregulation was observed after GT3 treatment. Significance counts and trends of up- and downregulation can be viewed for PBI and TBI groups in Table 1 and Table 2, respectively. Although there were some similarities in terms of dysregulated pathways and metabolites between PBI and TBI groups, some differential expression was also observed, with significant unique metabolites and pathways dysregulated or protected in either group. Additional figures including volcano plots for the 8 h time point to the d 6 time point can be viewed in Supplementary Figures S1–S5.

### 3.1. Effects of Partial-Body Radiation on Metabolomic Profiles

PBI induced significant metabolomic perturbations in irradiated animals. The number of significantly dysregulated metabolites reached its highest level 4 h after exposure to 12 Gy partial-body radiation in both the radiation and drug effects comparisons (Figure 2B,C). Alpha-pinene-oxide and octadecenoic acid were the most significantly dysregulated metabolites at the 4 h time point, but this dysregulation waned as the study progressed. Overall, dysregulation gradually decreased as the study progressed for both comparisons, but was increased slightly on d 6 in the radiation comparison. Interestingly, opposite directionality was observed between the radiation effects and GT3 effects comparisons, further confirming our hypothesis that GT3 would mitigate the irradiation-induced dysregulation on metabolomic profiles to some extent. A majority upregulation was observed in the radiation effects comparison during the hourly time points following irradiation, specifically at 4, 8, and 12 h, which then fluctuated throughout the remainder of the study. Conversely, in the drug effects comparison, a majority downregulation was observed in all time points analyzed, suggesting GT3 offered a modulatory effect on irradiation-induced dysregulation. Overall significance counts and directionality for each time point can be viewed in Table 1.

**Figure 3:**
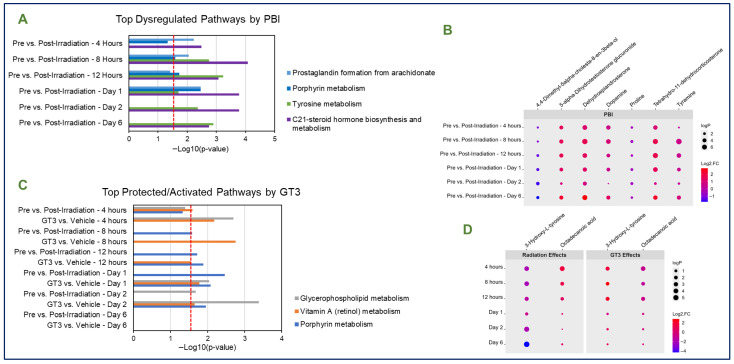
Top dysregulated pathways (**A**) and metabolites (**B**) in serum of NHPs exposed to partial-body radiation. (**C**) Top significant pathways in GT3-treated animals exposed to PBI. Notably, vitamin (A) retinol metabolism was significantly protected at 4 h and significantly activated in all time points apart from Day 6, while porphyrin metabolism was significantly protected by GT3 administration at the 12 h and Day 1 time points and glycerophospholipid metabolism was significantly protected at 4 h and day 2. (**D**) Top significant metabolites in GT3-treated animals exposed to PBI. Pathway analysis was performed using Mummichog.

Pathway and metabolite analysis were performed to assess the effects of PBI, which was found to induce significant dysregulation to several notable pathways and metabolites at several time points independent of GT3 administration. The C21-steroid hormone biosynthesis and metabolism, tyrosine metabolism, porphyrin metabolism, and prostaglandin from arachidonate pathways were the most commonly dysregulated pathways in partial-body irradiated animals (Figure 3A). Several notable metabolites including tyramine; proline; dopamine; Dehydroepiandrosterone; Tetrahydro-11-dehydrocorticosterone; 4,4-Dimethyl-5alpha-cholesta-8-en-3beta-ol; and 5alpha-Dihydrotestosterone glucuronide were significantly dysregulated in partial-body irradiated animals despite GT3 administration (Figure 3B).

Both comparisons were analyzed collectively to determine the protective effects afforded by administration of GT3 in partial-body irradiated animals. GT3 offered modest protection to a few select pathways (Figure 3C). Notably, dysregulation to the vitamin A (retinol) metabolism pathway was minimal in the radiation effects comparisons, but was significant in the GT3 effects comparisons at all time points except d 6. When comparing GT3-treated and vehicle-treated animals at the 4 h time point (“GT3 effects” comparison), this comparison was statistically significant, suggesting that GT3 administration protected this pathway. Other significantly dysregulated pathways including the porphyrin metabolism and glycerophospholipid metabolism and were significantly protected at varying isolated time points throughout the study, but were not consistently protected throughout the study. One metabolite, 4-oxo-9-cis-retinoyl-beta-glucuronide (a metabolite of vitamin A), was significantly upregulated at all time points analyzed. GT3 offered radioprotective effects to metabolites such as 3-Hydroxy-L-tyrosine, which was significantly protected only up until the d 1 time point. Octadecanoic acid was also significantly protected, but only until the 12 h time point. Raindrop plots were constructed to visualize the significance and directionality of these protected metabolites, which is displayed in Figure 3D.

### 3.2. Effects of Total-Body Radiation on Metabolomic Profiles

Similarly to partial-body irradiated animals, the highest degree of metabolic dysregulation was 4 h post TBI (Figure 2D,E). Notably, similar to the PBI group, octadecenoic acid was one of the most significantly dysregulated metabolites in both groups at the 4 h time point, but dysregulation was minimal throughout the remainder of the study. Thereafter, dysregulation in terms of significantly dysregulated metabolites gradually declined until the d 1 time point and plateaued for the remainder of the study. No consistent trend of up- or downregulation was observed in either comparison; a majority upregulation was observed at 4 h, 8 h, 2 d, and 6 d post-irradiation, while a majority downregulation was noted at the 12 h and d 1 time point. Overall significance counts and directionality for each time point in the TBI group can be viewed in Table 2.

**Figure 4:**
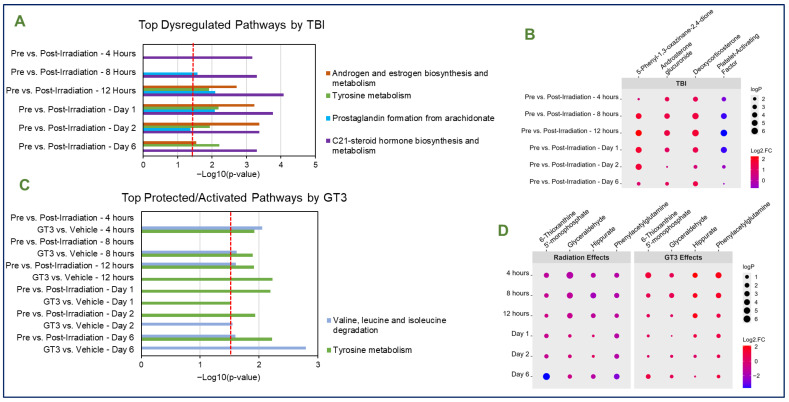
Top dysregulated pathways (**A**) and metabolites (**B**) in serum of NHPs exposed to total-body radiation. (**C**) Top significant pathways in GT3-treated animals exposed to TBI. Tyrosine metabolism was significantly protected at 12 h and Day 1, while the valine, leucine, and isoleucine pathway was significantly protected at Day 6. Notably, these pathways are also significant when comparing GT3-treated to vehicle-treated animals at various time points. (**D**) Top significant metabolites in GT3-treated animals exposed to TBI. Pathway analysis was performed using Mummichog.

Pathway and metabolomic analysis were conducted to evaluate the effects of TBI on metabolomic profiles in an effort to assess any similarities or differences in response compared to the PBI group. As observed in the PBI group, the most significantly dysregulated pathway by TBI was the C21-steroid hormone biosynthesis and metabolism. Other pathways including tyrosine metabolism, prostaglandin formation from arachidonate, and the androgen and estrogen biosynthesis and metabolism pathways were significantly dysregulated beginning around the 8–12 h time points and continuing to the d 2–d 6 time points. The top dysregulated pathways affected by TBI are detailed in Figure 4A. Notably, irradiation-induced dysregulation to metabolic pathways was highest at the d 2 time point. Consistent significance in several unique metabolites in the radiation effects comparison was noted in the TBI group. Metabolites including androsterone glucuronide, deoxycorticosterone, and 5-Phenyl-1,3-oxazinane-2,4-dione were consistently upregulated at all time points in all comparisons, while platelet-activating factor was consistently downregulated (Figure 4B).

The protective effects of GT3 were then assessed by comparing both the radiation effects and GT3 effects comparisons collectively. Two pathways were significant in response to GT3 treatment at various time points, as displayed in Figure 4C. For example, the valine, leucine, and isoleucine degradation pathway was minimally affected by radiation exposure, but significant in the GT3 effects comparison at a few time points. As this pathway was not directly dysregulated by radiation exposure, the significance of this pathway at several time points in GT3-treated animals suggests GT3 may offer radioprotection via pathways previously unknown. The tyrosine metabolism pathway was significantly protected by GT3 administration at the 12 h and d 1 time points. A few of the metabolites followed unique trends throughout the course of the study (Figure 4D). For example, betaine was significantly downregulated at a majority of time points analyzed in both the radiation effects and GT3 effects comparisons. Only one metabolite, phenylacetylglutamine, was consistently and significantly protected by GT3 administration at all time points analyzed. Other significantly protected metabolites were time-dependent; hippurate was significantly protected up until the d 1 time point, while glyceraldehyde was significantly protected up until the 12 h time point. Lastly, 6-Thioxanthine 5′-monophosphate was significantly protected at 4 h, 8 h, 12 h, and d 6. Overall, the protective effects of GT3 were modest, but similar in terms of the extent of affected pathways to those observed in the PBI group.

### 3.3. Metabolic Patterns Are Consistent Across Radiation Types

**Figure 5:**
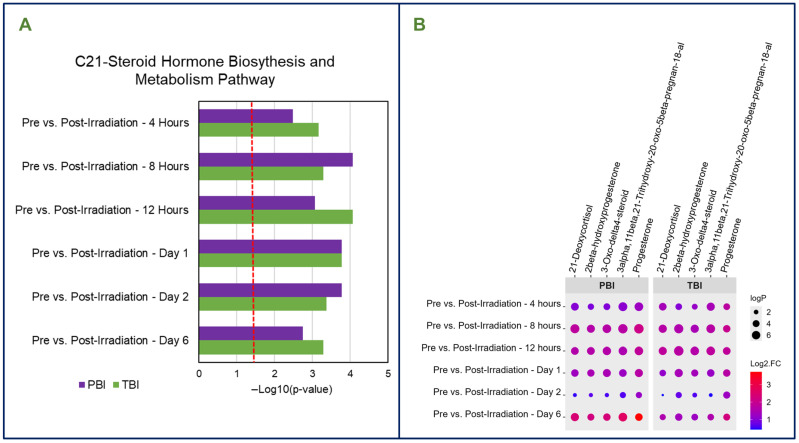
(**A**) Consistent dysregulation to the C-21 steroid hormone biosynthesis and metabolism pathway was observed in serum samples collected from both partial- and total-body irradiated animals. (**B**) Steroid metabolites significantly dysregulated by both PBI and TBI.

Although a greater degree of dysregulation in terms of significantly dysregulated metabolites was expected in the PBI group due to bone marrow sparing, we observed an overlap in terms of dysregulated metabolites and pathways between the PBI and TBI groups. Both radiation groups generally followed similar trends in terms of the number of significantly dysregulated metabolites as well as the trend of directionality of metabolites. Interestingly, when comparing the post-irradiation time points to the pre-irradiation time points, a majority upregulation was noted in these comparisons, while a majority downregulation was observed in the GT3 comparisons, suggesting GT3 offered a modulatory effect on irradiation-induced dysregulation in both PBI and TBI animals. Several metabolites were consistently dysregulated by radiation exposure, regardless of radiation exposure type or GT3 treatment, including 3-Oxo-delta4-steroid; progesterone; 3alpha,11beta,21-Trihydroxy-20-oxo-5beta-pregnan-18-al; 2beta-hydroxyprogesterone; and 21-Deoxycortisol. Notably, many of these metabolites play a role in the C21-steroid hormone biosynthesis and metabolism pathway, which was the most significantly dysregulated pathway in both PBI and TBI animals (Figure 5A,B). Other pathways including tyrosine metabolism as well as prostaglandin formation from arachidonate were significantly dysregulated by either PBI or TBI, but to varying degrees.

GT3 afforded a variable radioprotective effect that was determinant on radiation exposure type to a few notable pathways and metabolites in both PBI and TBI groups. Somewhat unexpectedly, minimal overlap was observed in terms of significantly protected metabolites or pathways between PBI and TBI groups. In the PBI group, dysregulation to the porphyrin metabolism pathway was alleviated by GT3 administration at 12 h and d 1 post-irradiation. Two metabolites that participate in this pathway, dopamine and tyramine, were significantly protected by GT3 administration at varying time points throughout the study. In the TBI group, the tyrosine metabolism pathway was significantly protected 12 h and d 1 post-irradiation. In both the PBI and TBI groups, the glycerophospholipid metabolism pathway was significant. However, it is important to note that these pathways were not consistently dysregulated solely by type of radiation exposure and were only consistently significant in the drug effects comparisons.

## 4. Discussion

Metabolomics is an emerging field of systems biology that we hope to leverage in our efforts to develop radiation MCMs which can be used in various nuclear events to protect emergency responders against the deleterious effects of radiation exposure. In doing so, our understanding of the effects of ionizing radiation on a molecular level will augment translation into other areas of pharmaceutical development. Our laboratory has previously investigated metabolomic changes in serum and tissue samples of animals exposed to Cobalt-60 gamma (^60^Co γ) total-body radiation, including murine and NHP models. Studies using NHP models were performed with both 4 and 5.8 Gy total-body γ-radiation and partial-body X-ray radiation. Additionally, metabolomic studies have been performed with several radiation MCMs that are being developed for prophylactic use in our lab including amifostine, BIO 300, Ex-Rad, and GT3. Amifostine studies were completed in a murine model while Ex-Rad, BIO 300, and GT3 studies were completed in an NHP model [33].

Several tissue types were collected from the same 32 animals used in this study, and various analyses were performed to assess the effects of both radiation exposure and GT3 efficacy. Transcriptomic analysis was performed on both lung and jejunum tissue in NHPs with PBI or TBI. Significant irradiation-induced changes were observed in the lung transcriptome for total-body exposed animals [39]. However, there was no major effect of GT3 on irradiation-induced transcriptomic changes observed, demonstrating that this potential MCM may not be effective in protecting against a supralethal dose of radiation. Regardless, several signaling pathways such as growth arrest and DNA damage-inducible (GADD45), p35, and Phosphoinositide 3-kinase/Protein Kinase B (PI3K/AKT) pathway were upregulated following total-body radiation exposure. Similar irradiation-induced effects were observed in jejunum tissue collected from animals exposed to partial- or total-body radiation. Focal adhesion kinase (FAK) signaling, cAMP response element-binding protein (CREB) signaling in the neurons, phagosome formation, and G-protein coupled signaling pathways were found to be activated following PBI and TBI [38]. However, as was observed in lung tissue, GT3 did not have a major effect on the transcriptome at such a high dose of radiation. Additionally, histopathological studies were performed to assess GT3-mediated GI injury in TBI or PBI NHPs. Plasma citrulline, crypt cell proliferation and apoptotic cell death, mucosal surface area, and villous height were also investigated in PBI animals, and our data suggests that 12 Gy X-rays induce severe intestinal injury post-PBI and GT3 provided limited radioprotection against such a high dose [35]. In TBI animals, there were significant decreases in mucosal surface area and villous height, as observed in the partial body exposed animals. GT3 prophylaxis induced an increase in crypt depth and Ki-67-positive cells and caused a decline in TUNEL-positive cells in intestinal tissue of animals exposed to total-body radiation [36]. Ultimately, based on our observations in these previous studies, GT3 seems capable of affording pronounced radioprotection in sublethal radiation doses, while more modest protection is observed in supralethal radiation doses.

This study compares the metabolomic serum profiles of male and female NHPs exposed to a single supralethal radiation dose of either 12 Gy PBI or TBI. Additionally, we also assessed the ability of GT3 to protect against the deleterious effects of radiation on metabolomic profiles. Irradiation induced similar effects in either PBI or TBI group, with dysregulation peaking at 4 h post-irradiation and gradually declining throughout the course of the study. Interestingly, dysregulation was slightly greater in the PBI group, while the opposite was expected. In assessing the effects of GT3, a similar trend was noted, where more dysregulation/protection was observed in the PBI group compared to the TBI group. Regardless, GT3 offered significant metabolomic protection against radiation injury in both the PBI and TBI groups and gradually decreased as the study progressed.

Both PBI and TBI were found to induce significant inflammation in irradiated animals, as observed by dysregulation to the C21-steroid hormone biosynthesis and metabolism pathway. A few C21-steroid hormones were found to be consistently and significantly dysregulated in both PBI and TBI groups despite GT3 administration, including 21-Deoxycortisol, 2beta-hydroxyprogesterone, 3-Oxo-delta4-steroid, 3alpha,11beta, 21-Trihydroxy-20-oxo-5beta-pregnan-18-al, and progesterone. Previous studies have consistently shown dysregulation in this pathway in both serum and tissue samples in animals exposed to either partial- or total-body radiation [45,46,47,48,49]. This pathway is proven to be highly sensitive to radiation exposure, both directly and indirectly. Not only is this pathway crucial for the production of cortisol and other important C21-steroid hormones such as aldosterone, this pathway helps regulate cellular stress and inflammation. Of specific interest, the presence of progesterone among the dysregulated metabolites could indicate an effect of radiation on sex hormones and an impact on the reproductive or endocrine systems.

Minimal protective effects of GT3 were observed in either PBI or TBI group. A few pathways were significantly protected by GT3 at a few isolated time points; however, this protective effect was not consistent throughout the course of the study. In the PBI group, porphyrin metabolism was protected at 12 h and d 1, while the glycerophospholipid metabolism pathway was protected at 4 h and d 2. Notably, the vitamin (A) retinol metabolism pathway was only significantly protected at 4 h; however, this pathway was significant in the GT3 effects comparisons at all time points apart from d 6. As this pathway is related to oxidative stress, this significance indicates that this pathway may be stimulated by the administration of GT3 due to its antioxidant activity. For TBI, the tyrosine metabolism pathway was significantly protected at 12 h and d 1, while the valine, leucine, and isoleucine degradation pathway was significant only at d 6. As with the PBI comparisons, these pathways were also significant in the GT3 effects comparison in TBI animals. The protection of certain metabolites such as dopamine and tyramine in the tyrosine metabolism pathway suggests that GT3 may offer a wider arc of protective effects that extends to neurotransmitters and neuromodulators by neutralizing reactive oxygen species produced by ionizing radiation exposure. These results suggest GT3 affords radioprotection via its antioxidant activity by scavenging free radicals and reducing radiation-induced oxidative stress.

Ultimately, our results suggest that GT3 is capable of affording modest metabolomic protection against supralethal doses of 12 Gy PBI or TBI. However, this protection is short-lived, and the radioprotective effects of GT3 dwindle as the time post-irradiation increases. Similar trends were observed in irradiated animals, regardless of the type of exposure. Ionizing radiation drives the generation of reactive oxygen species (ROS), which can oxidize lipids, proteins, and DNA, thereby disrupting normal enzymatic function and altering metabolite pools. For example, ROS-mediated oxidative damage to mitochondria and endoplasmic reticulum can impair steroidogenic enzymes, leading to significant dysregulation of the C21-steroid hormone biosynthesis and metabolism pathway in both PBI and TBI groups; this suggests that perturbation of key hydroxysteroid dehydrogenases and cytochrome P450 enzymes underpins the observed hormonal imbalance. The well-documented irradiation-induced imbalance to this hormonal pathway must be investigated further to fully understand the complex molecular changes caused by ionizing radiation exposure. Differential metabolomic response attributed to GT3 administration was observed in terms of protected pathways and metabolites when comparing PBI to TBI. This response could be attributed to the 5% bone marrow sparing afforded to the PBI group. Many of the pathways affected by radiation exposure and protected by GT3 administration are related to oxidative stress; radiation-induced ROS can inactivate thiol-dependent enzymes and deplete cellular glutathione, leading to downstream metabolite dysregulation. GT3’s potent antioxidative effects, such as direct free radical scavenging via its chromanol ring and upregulation of endogenous antioxidant defenses (e.g., induction of glutamate–cysteine ligase and superoxide dismutase expression), help restore redox homeostasis and protect sensitive metabolites [50]. Additionally, a few protected pathways were related to amino acid metabolism, suggesting GT3 may afford protective effects to pathways previously unknown.

There are some limitations of this study which must be considered. The metabolite identifications discussed herein are putative, and validation will need to be performed in the future to confirm compound identities using tandem mass spectrometry of the unknown compounds and a known standard compound against the National Institute of Standards and Technology spectral database. Due to the supralethal dose of radiation used, animal health declined very quickly, and all animals were moribund and humanely euthanized by d 7 post-irradiation. Therefore, our observations in this study are limited to early radiation-induced metabolomic changes and early protective effects of GT3. Considering the high lethality observed with 12 Gy, somewhat lower doses of radiation may be used in future studies. GT3 is highly effective against H-ARS and, based on this study, we assume GT3 will have limited efficacy against GI-ARS induced by high doses of radiation. As with all studies, confounding factors that could potentially influence the interpretation of results should be considered. One possible confounding factor is that the rhesus macaques are outbred, which introduces greater genetic variability. As a result, biological responses to stress from being handled for blood collections, irradiation, drug treatment, etc., could differ between animals. Additional studies should be performed with varying doses and other radiation sources including low and high linear energy transfer (LET) and mixed field (neutron/gamma) to assess metabolomic changes induced by radiation exposure and pathways whereby GT3 exerts its radioprotective efficacy. Notably, the difference in dose rates between PBI (1.3 Gy/min) and TBI (0.6 Gy/min) that was required for experimental conditions may have contributed to some of the observed differences between these groups. In the future, work will also be needed to investigate the long-term effects of GT3 and to evaluate its efficacy when treatment is combined with antibiotics or with other combination therapies under development [51]. Lastly, integrated analysis of transcriptomics, metabolomics, and proteomics data should be performed to gain valuable insight into radiation-induced disease progression and the mechanism of action of GT3, ultimately providing a broader understanding of these biological mechanisms and guiding future research. We plan to accomplish such analyses in the future.

## Figures and Tables

**Table 1:** An overall count of the putatively identified significantly dysregulated metabolites as well as overall trend in directionality in partial-body irradiated animals.

Radiation Group	Time Point	Total Significant Metabolites (*p*-Value)	Metabolites (↑ | ↓)	Total Significant Metabolites (FDR)	Metabolites (↑ | ↓)
PBI	Pre vs. Post-Irradiation (Vehicle-Treated)—4 h	134	(105 | 29)	122	(99 | 23)
	Pre vs. Post-Irradiation (Vehicle-Treated)—8 h	102	(88 | 14)	58	(55 | 3)
	Pre vs. Post-Irradiation (Vehicle-Treated)—12 h	91	(55 | 36)	58	(35 | 23)
	Pre vs. Post-Irradiation (Vehicle-Treated)—Day 1	72	(38 | 34)	40	(25 | 15)
	Pre vs. Post-Irradiation (Vehicle-Treated)—Day 2	45	(24 | 21)	11	(6 | 5)
	Pre vs. Post-Irradiation (Vehicle-Treated)—Day 6	73	(53 | 20)	53	(39 | 14)
	GT3-Treated vs. Vehicle-Treated—4 h	91	(17 | 74)	4	(1 | 3)
	GT3-Treated vs. Vehicle-Treated—8 h	67	(10 | 57)	3	(1 | 2)
	GT3-Treated vs. Vehicle-Treated—12 h	51	(14 | 37)	3	(3 | 0)
	GT3-Treated vs. Vehicle-Treated—Day 1	36	(15 | 21)	0	(0 | 0)
	GT3-Treated vs. Vehicle-Treated—Day 2	34	(7 | 27)	0	(0 | 0)
	GT3-Treated vs. Vehicle-Treated—Day 6	21	(2 | 19)	0	(0 | 0)

FDR: false discovery rate, ↑: upregulated metabolites, ↓: downregulated metabolites.

**Table 2:** An overall count of the putatively identified significantly dysregulated metabolites as well as overall trend in directionality in total-body irradiated animals.

Radiation Group	Time Point	Total Significant Metabolites (*p*-Value)	Metabolites (↑ | ↓)	Total Significant Metabolites (FDR)	Metabolites (↑ | ↓)
TBI	Pre vs. Post-Irradiation (Vehicle-Treated)—4 h	115	(88 | 27)	70	(57 | 13)
	Pre vs. Post-Irradiation (Vehicle-Treated)—8 h	86	(41 | 45)	53	(25 | 28)
	Pre vs. Post-Irradiation (Vehicle-Treated)—12 h	66	(32 | 34)	52	(28 | 24)
	Pre vs. Post-Irradiation (Vehicle-Treated)—Day 1	57	(37 | 20)	37	(27 | 10)
	Pre vs. Post-Irradiation (Vehicle-Treated)—Day 2	63	(53 | 10)	5	(4 | 1)
	Pre vs. Post-Irradiation (Vehicle-Treated)—Day 6	60	(36 | 24)	27	(17 | 10)
	GT3-Treated vs. Vehicle-Treated—4 h	74	(34 | 40)	5	(5 | 0)
	GT3-Treated vs. Vehicle-Treated—8 h	54	(52 | 2)	2	(2 | 0)
	GT3-Treated vs. Vehicle-Treated—12 h	39	(14 | 25)	1	(0 | 1)
	GT3-Treated vs. Vehicle-Treated—Day 1	23	(4 | 19)	0	(0 | 0)
	GT3-Treated vs. Vehicle-Treated—Day 2	72	(8 | 64)	19	(0 | 19)
	GT3-Treated vs. Vehicle-Treated—Day 6	13	(10 | 3)	0	(0 | 0)

FDR: False discovery rate, ↑: upregulated metabolites, ↓: downregulated metabolites.

## Data Availability

All relevant data that supports the findings of the study are within the manuscript and Supplementary Files.

## Supplementary Materials

The following supporting information can be downloaded at: https://www.mdpi.com/article/10.3390/metabo15080546/s1, Figure S1: Volcano plots comparing NHP serum profiles 8 h post partial-body irradiation (A,B) or post total-body irradiation (C,D); Figure S2: Volcano plots comparing NHP serum profiles 12 h post partial-body irradiation (A,B) or post total-body irradiation (C,D); Figure S3:Volcano plots comparing NHP serum profiles 1 day post partial-body irradiation (A,B) or post total-body irradiation (C,D); Figure S4: Volcano plots comparing NHP serum profiles 2 days post partial-body irradiation (A,B) or post total-body irradiation (C,D); Figure S5: Volcano plots comparing NHP serum profiles 6 days post partial-body irradiation (A,B) or post total-body irradiation (C,D); Table S1: An exhaustive list of metabolites screened for in the serum samples collected from partial-body irradiated animals; Table S2: An exhaustive list of metabolites screened for in the serum samples collected from total-body irradiated animals; Table S3:Metabolomics data of serum collected from partial-body irradiated animals comparing pre-irradiation and post-irradiation samples (vehicle-treated animals) to assess the effects of radiation, as well as between samples collected from GT3-treated animals and vehicle-treated animals at post-irradiation time points to assess the effects of GT3; Table S4:Metabolomics data of serum collected from total-body irradiated animals comparing pre-irradiation and post-irradiation samples (vehicle-treated animals) to assess the effects of radiation, as well as between samples collected from GT3-treated animals and vehicle-treated animals at post-irradiation time points to assess the effects of GT3; Table S5: Pathway analysis results to assess the effects of partial-body irradiation or GT3 treatment at various time points; Table S6: Pathway analysis results to assess the effects of total-body irradiation or GT3 treatment at various time points.
