# Supplementary material for: Serum Metabolomic Signatures in Nonhuman Primates Treated with a Countermeasure and Exposed to Partial- or Total-Body Radiation
Source: Metabolites. 2025 Aug 12;15(8):546. doi: 10.3390/metabo15080546 (PMC12388117; doi:10.3390/metabo15080546)
Supplement: Supplementary file 1 [file metabolites-15-00546-s001.zip › metabolites-3796480-supplementary Figures S1-S5.pdf]

Supplementary Figures

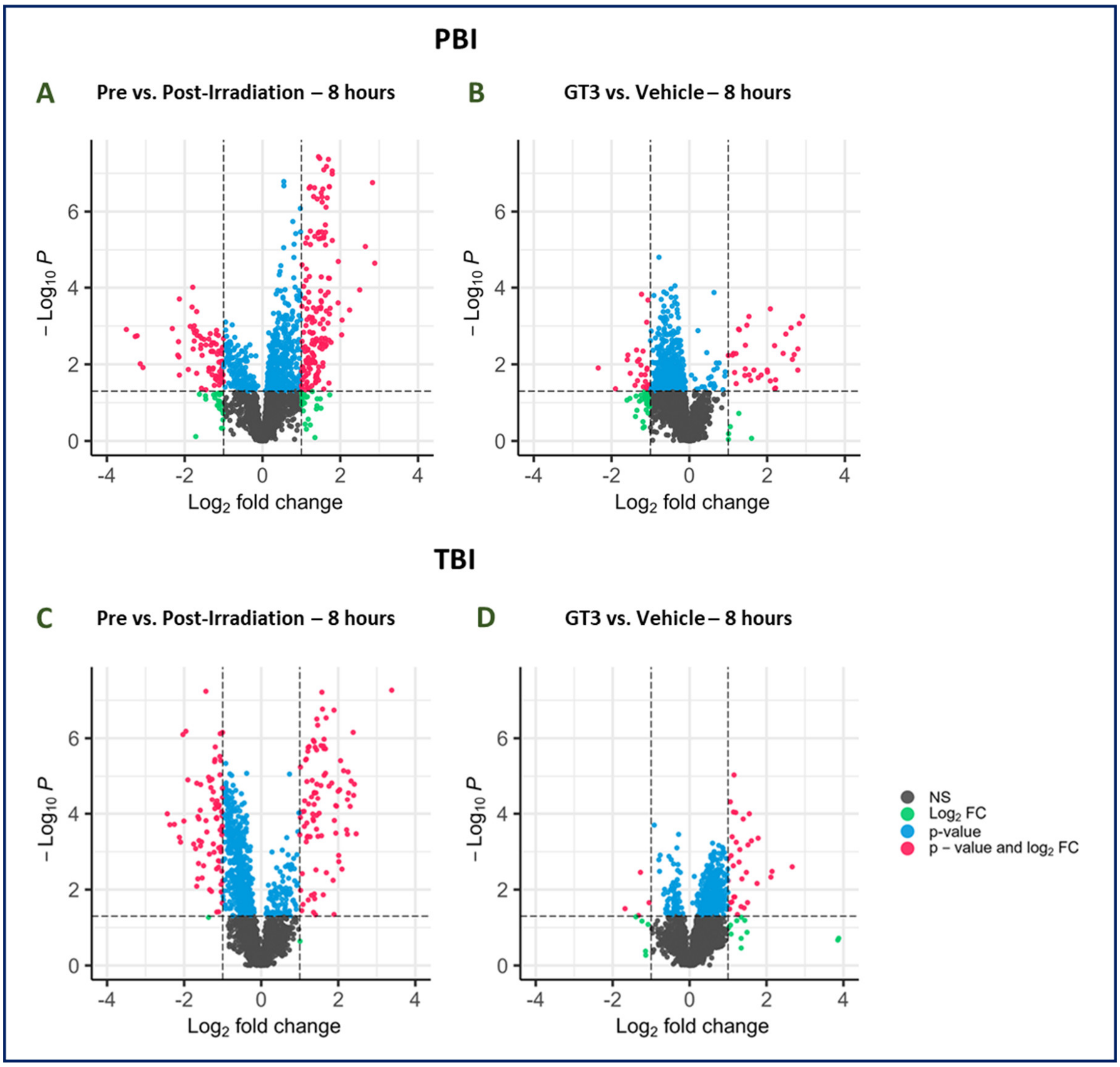

Supplementary Figure S1. Volcano plots comparing NHP serum profiles 8 hours post partial-body irradiation (**Panels A and B**) or post total-body irradiation (**Panels C and D**).

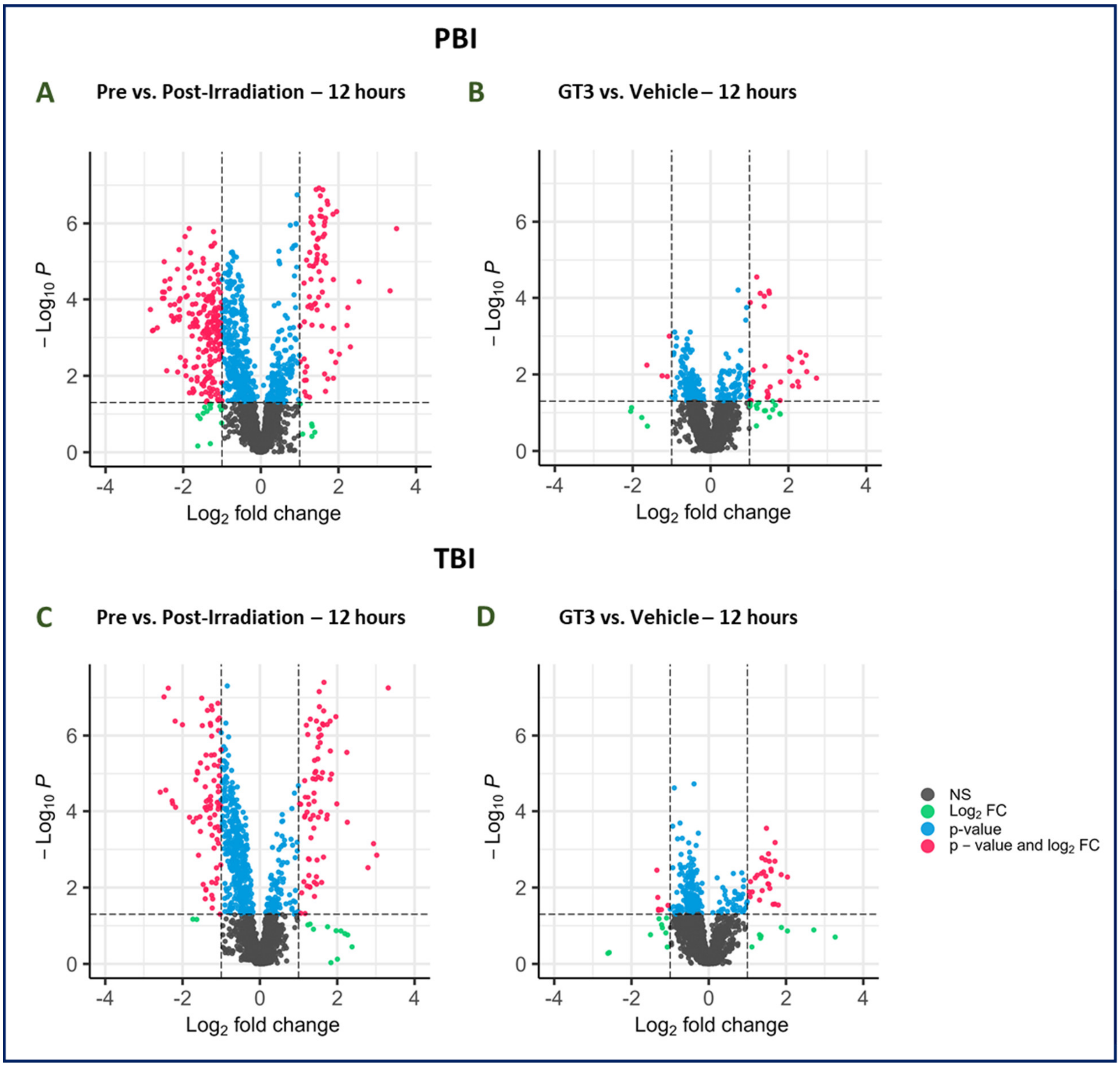

Supplementary Figure S2. Volcano plots comparing NHP serum profiles 12 hours post partial-body irradiation (**Panels A and B**) or post total-body irradiation (**Panels C and D**).

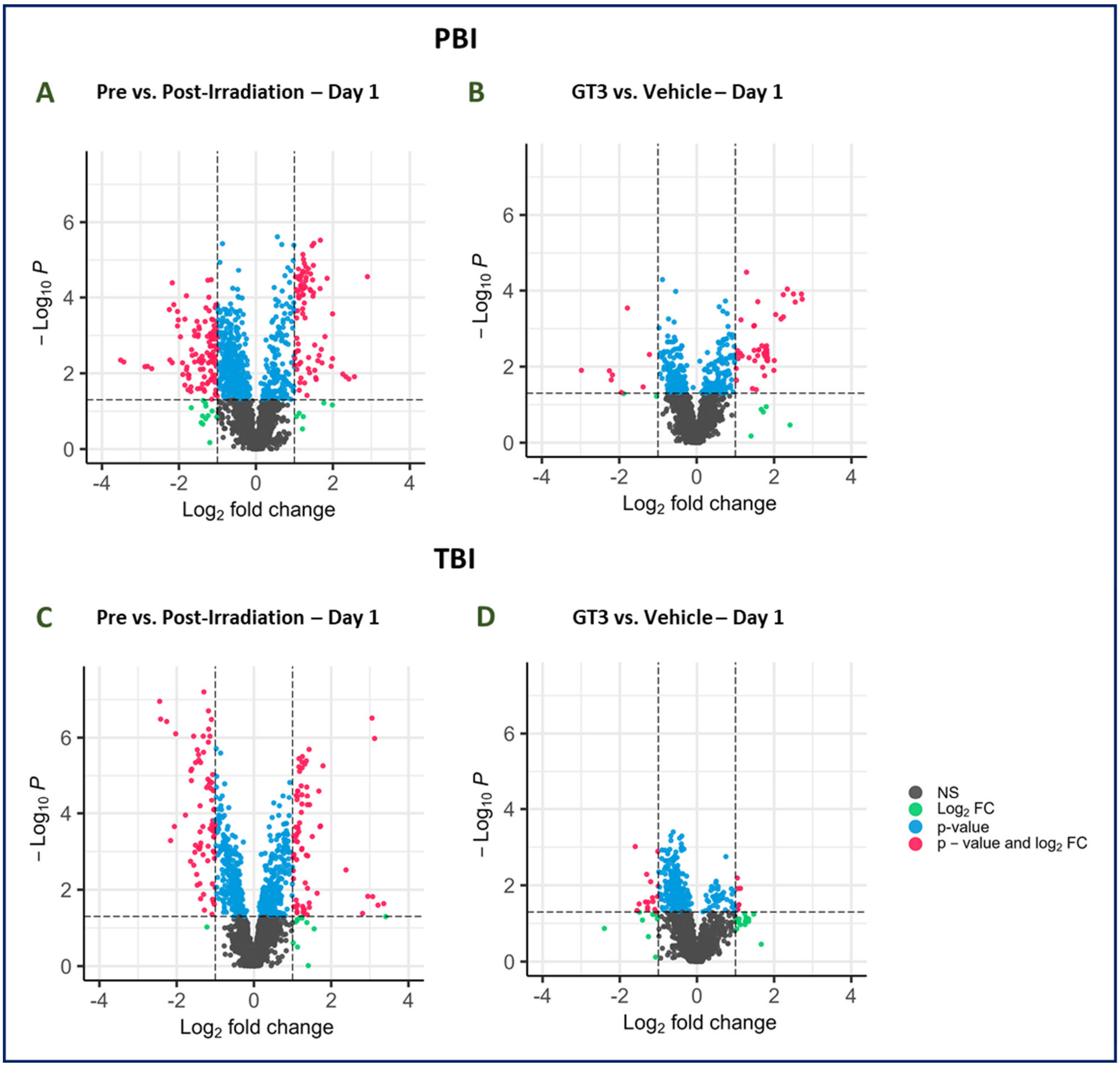

Supplementary Figure S3. Volcano plots comparing NHP serum profiles 1 day post partial-body irradiation (**Panels A and B**) or post total-body irradiation (**Panels C and D**).

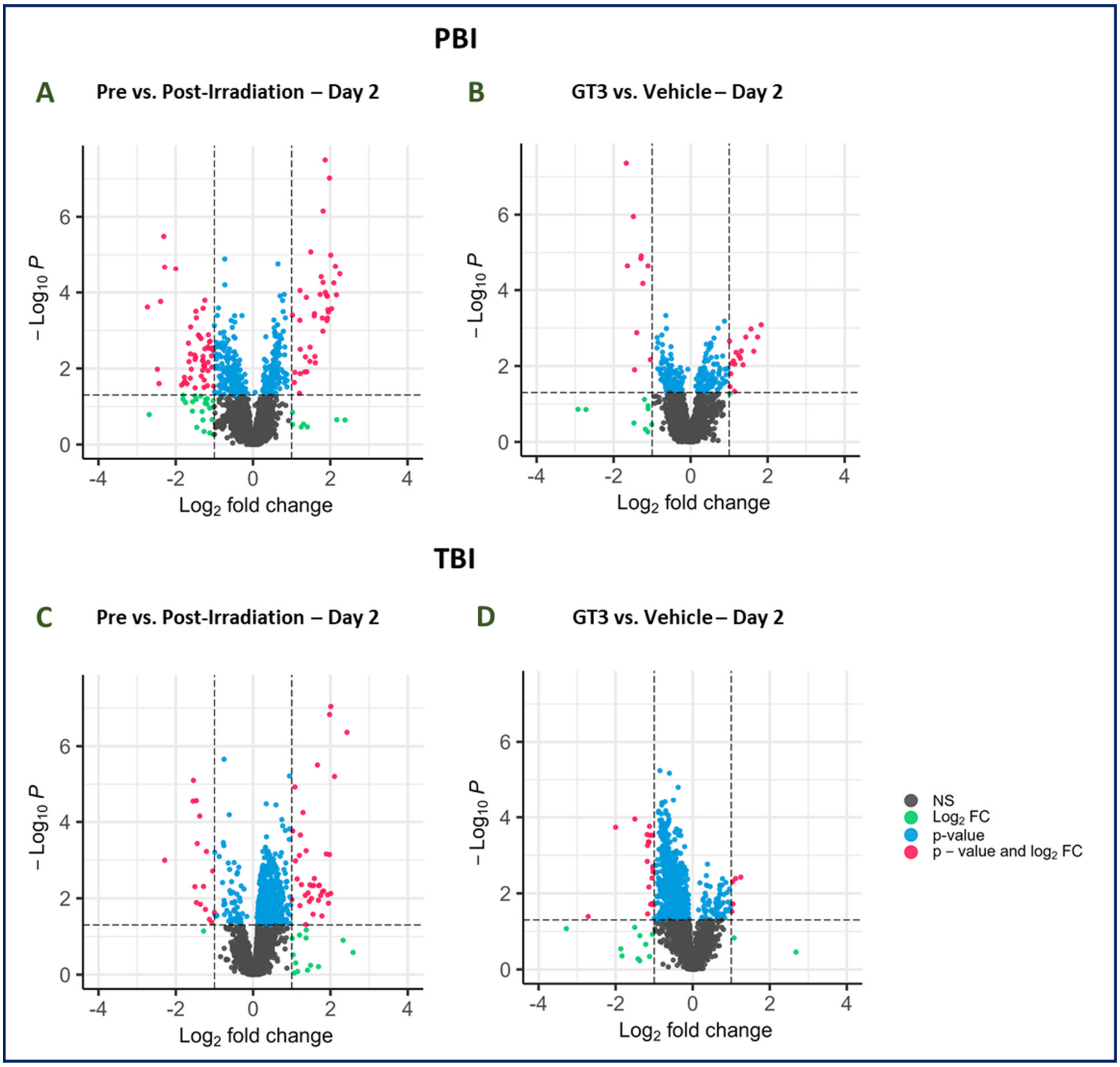

Supplementary Figure S4. Volcano plots comparing NHP serum profiles 2 days post partial-body irradiation (**Panels A and B**) or post total-body irradiation (**Panels C and D**).

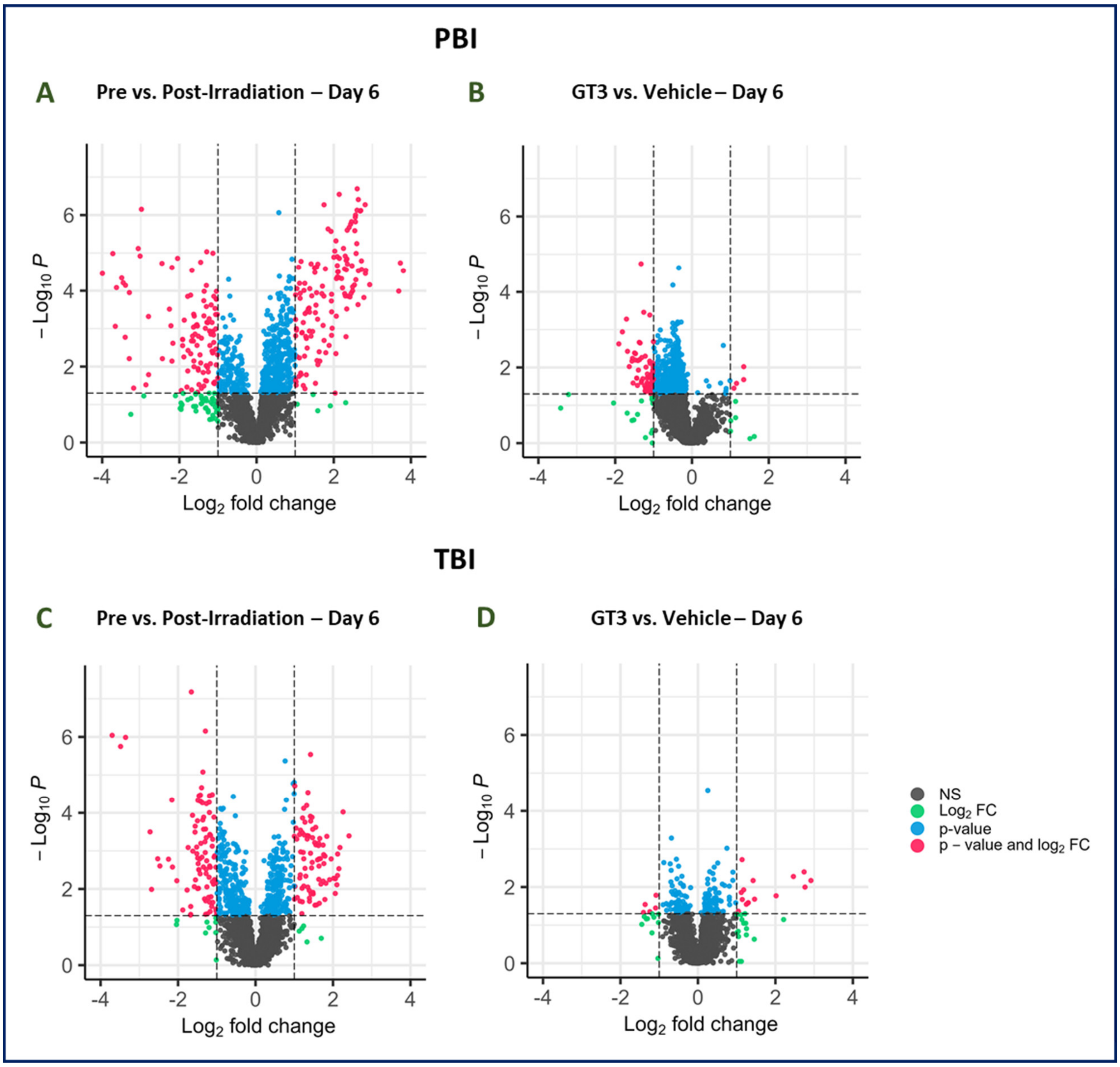

Supplementary Figure S5. Volcano plots comparing NHP serum profiles 6 days post partial-body irradiation (**Panels A and B**) or post total-body irradiation (**Panels C and D**).
